# Spheroid Model of Mammary Tumor Cells: Epithelial–Mesenchymal Transition and Doxorubicin Response

**DOI:** 10.3390/biology13070463

**Published:** 2024-06-21

**Authors:** Laura Lacerda Coelho, Matheus Menezes Vianna, Debora Moraes da Silva, Beatriz Matheus de Souza Gonzaga, Roberto Rodrigues Ferreira, Ana Carolina Monteiro, Adriana Cesar Bonomo, Pedro Paulo de Abreu Manso, Marcelo Alex de Carvalho, Fernando Regla Vargas, Luciana Ribeiro Garzoni

**Affiliations:** 1 Laboratory of Innovations in Therapies, Education and Bioproducts, Oswaldo Cruz Institute (IOC), Oswaldo Cruz Foundation (Fiocruz), Rio de Janeiro 21040-900, Brazil; llacerdac@gmail.com (L.L.C.); mtmenezesv@gmail.com (M.M.V.); dbmoraes.m@gmail.com (D.M.d.S.); biagonzaga04@hotmail.com (B.M.d.S.G.); robertoferreira@ioc.fiocruz.br (R.R.F.); 2 Laboratory of Osteo and Tumor Immunology, Department of Immunobiology, Fluminense Federal University (UFF), Rio de Janeiro 24020-150, Brazil; anacarolinadossantosmonteiro@id.uff.br; 3 Thymus Research Laboratory, Oswaldo Cruz Institute (IOC), Oswaldo Cruz Foundation (Fiocruz), Rio de Janeiro 21040-900, Brazil; acbonomo@gmail.com; 4 Laboratory of Pathology, Oswaldo Cruz Institute (IOC), Oswaldo Cruz Foundation (Fiocruz), Rio de Janeiro 21040-900, Brazil; mansoppa@gmail.com; 5 Research Center (CPQ), National Cancer Institute (INCA), Rio de Janeiro 20231-050, Brazil; marcelo.carvalho@inca.gov.br; 6 Laboratory of Epidemiology of Congenital Malformations, Oswaldo Cruz Institute (IOC), Oswaldo Cruz Foundation (Fiocruz), Rio de Janeiro 21040-900, Brazil; fernandorevargas@gmail.com

**Keywords:** breast cancer, three-dimensional cell culture, spheroids, doxorubicin, cell migration, epithelial–mesenchymal transition

## Abstract

**Simple Summary:**

Breast cancer is the type of cancer that most affects women worldwide, and until today, it is difficult to find an effective treatment against this disease. Scientists are exploring new ways to test treatments using systems in the laboratory capable of mimicking tumors, called 3D models or spheroids. This study aimed to understand how breast cancer spheroids behave and respond to a common drug used in the clinic to treat cancer, doxorubicin. Understanding these processes could lead to improved treatments for breast cancer and other types of cancer. We found that the spheroids showed close features of real tumors and showed changes in proteins associated with cancer spread (metastasis). When the spheroids were treated with doxorubicin, the size of the spheroids was reduced, cells died, and the spread of breast cancer cells was also reduced. These results suggest, for the first time, that doxorubicin could be a good candidate to help stop cancer metastasis, which can be further studied.

**Abstract:**

Breast cancer is the most prevalent cancer among women worldwide. Therapeutic strategies to control tumors and metastasis are still challenging. Three-dimensional (3D) spheroid-type systems more accurately replicate the features of tumors in vivo, working as a better platform for performing therapeutic response analysis. This work aimed to characterize the epithelial–mesenchymal transition and doxorubicin (dox) response in a mammary tumor spheroid (MTS) model. We evaluated the doxorubicin treatment effect on MCF-7 spheroid diameter, cell viability, death, migration and proteins involved in the epithelial–mesenchymal transition (EMT) process. Spheroids were also produced from tumors formed from 4T1 and 67NR cell lines. MTSs mimicked avascular tumor characteristics, exhibited adherens junction proteins and independently produced their own extracellular matrix. Our spheroid model supports the 3D culturing of cells isolated from mice mammary tumors. Through the migration assay, we verified a reduction in E-cadherin expression and an increase in vimentin expression as the cells became more distant from spheroids. Dox promoted cytotoxicity in MTSs and inhibited cell migration and the EMT process. These results suggest, for the first time, that this model reproduces aspects of the EMT process and describes the potential of dox in inhibiting the metastatic process, which can be further explored.

## 1. Introduction

Breast cancer is the most prevalent cancer among women worldwide, affecting about 2.3 million women each year [1]. The high mortality rate is mainly due to the occurrence of metastasis. Ductal and lobular invasive carcinomas are the most prevalent types of breast cancer and are associated with poor patient prognoses [2,3,4].

Metastasis is an important parameter associated with tumor progression, a multistep process wherein tumor cells can reach long distances in the body through blood or lymphatic vessels and colonize other tissues. Indeed, it is considered a hallmark of cancer [5,6,7]. An important initial step of metastasis is the capacity of cells to lose epithelial features and acquire a mesenchymal phenotype, conferring migratory and invasive properties to tumor cells in a process known as the epithelial–mesenchymal transition (EMT) [8,9,10,11]. A key feature of this process involves the loss of E-cadherin (E-cad) protein, an adherens junction protein [12,13], and an increase in vimentin (VIM) expression, a cytoskeleton component, by mesenchymal cells [9,14,15]. In addition, breast tumor cells can also migrate collectively (in groups) or through amoeboid movements [16,17,18,19].

Major scientific and technological advances have led to the discovery of new subtypes, mutations, and breast cancer biomarkers and contributed to a better understanding of tumoral behavior. These discoveries enabled the emergence of novel therapeutic advancements, such as targeted therapy and immunotherapy [20,21], which have improved the overall survival and disease progression rates [22,23,24]. However, therapeutic agents that inhibit the metastatic process are still challenging [25].

The three-dimensional (3D) systems of cell culture better mimic the tumor architecture and behavior, responding to in vitro treatment more similarly to in vivo treatment than traditional two-dimensional (2D) cell culture systems [26,27,28,29]. Within the 3D culture, spheroids are the most widely used type for cancer research, including breast cancer, once they can recapitulate the architecture, molecular, and functional characteristics of tumors in vivo [30,31,32,33,34]. Furthermore, large spheroids (200 to 500 μm in diameter) can reproduce avascular tumors and micrometastasis heterogeneity, characterized by a necrotic core and actively proliferative cells at the periphery [35,36]. Moreover, spheroids are considered the best model for performing high-throughput drug screening [37,38,39] and can also be used for migration and invasion analysis. For example, some studies assessed the invasive or migratory potential of tumor cells using traditional transwell systems [40,41]. However, in 2015, a 3D model was patented using reversed spheroids produced by ultra-low attachment conditions in six-well plates that reproduced metastasis features in vivo [42].

Previous studies of our group using multiple non-tumoral cell type spheroids [43,44,45,46] indicate that they are functional and capable of producing vascular structures when co-cultured with endothelial cells [44,45]. They are also able to generate a complex network of extracellular matrix and reproduce cardiac tissue fibrosis under stimulus, responding to distinct drug treatments [47,48].

In this study, we aimed to characterize the epithelial–mesenchymal transition and doxorubicin response in a mammary tumor spheroid (MTS) model. Our results revealed that dox promoted cytotoxicity in MTSs, reduced cell migration and inhibited the changes observed in E-cadherin and vimentin expression, characteristic of the EMT process. These in vitro results suggest, for the first time, the potential of dox in inhibiting the metastatic process, which can be further explored.

## 2. Materials and Methods

### 2.1. Reagents and Antibodies

Trypsin and ethylenediaminetetraacetic acid (EDTA) were acquired from Gibco (Carlsbad, CA, USA). Doxorubicin hydrochloride (DOX HCl), agarose, phosphate-buffered saline (PBS), fetal bovine serum (FBS), penicillin, streptomycin, RPMI 1640 and bovine serum albumin (BSA) were obtained from Sigma-Aldrich (St. Louis, MO, USA). Primary antibodies, rabbit polyclonal antibodies anti-fibronectin and anti-laminin, mouse monoclonal antibody anti-vimentin, and rabbit monoclonal antibody anti-E-cadherin were obtained from Sigma-Aldrich. Rabbit monoclonal antibody anti-Ki-67 was obtained from ABCAM (Cambridge, MA, USA), and mouse monoclonal antibody anti-GAPDH was acquired from Fitzgerald (Acton, MA, USA). Secondary antibodies, goat anti-rabbit and goat anti-mouse IgG Alexa Fluor 488 were purchased from Thermo Scientific (Rockford, IL, USA). Goat anti-rabbit IgG and goat anti-mouse IgG HRP-labeled were obtained from R&D Systems (Minneapolis, MN, USA). 7-AAD Ready-Made Solution was purchased from BD Bioscience (Franklin Lakes, NJ, USA). BCA protein assay reagent (bicinchoninic acid), 4′-6-Diamidino-2-phenylindole (DAPI), and ProLong™ Gold Antifade Mountant were acquired from Thermo Scientific. The Protease Inhibitor Cocktail was purchased from Roche Molecular Biochemicals (Indianapolis, IN, USA), the phosphatase inhibitor cocktail from Sigma Aldrich, and the chemiluminescent kit ECL from Pierce (Rockford, IL, USA).

### 2.2. Spheroid Formation Using Cell Line and Primary Culture of Mouse Tumor Cells

The human breast cancer cell line MCF-7 (ATCC^®^ HTB-22™) was obtained from the National Cancer Institute in Rio de Janeiro, Brazil. The cells were propagated as monolayers (2D) before three-dimensional (3D) cell culture. To allow the formation of multicellular tumor spheroids, or simply spheroids, cells in a monolayer were dissociated with trypsin/EDTA in HBSS without calcium and magnesium. The isolated cells were plated at different densities of 3125 to 25,000 cells per well in agarose-coated 96-U-well plastic plates [46,47]. Spheroids were maintained at 37 °C in a 5% CO_2_ atmosphere in RPMI 1640 supplemented with 10% FBS, 1000 U/mL penicillin and 50 μg/mL streptomycin. For the migration assay, spheroids were transferred to a 24-well plastic plate without any scaffold. The 4T1 and 67NR mouse mammary tumors were obtained from the Thymus Research Laboratory at the Oswaldo Cruz Institute (IOC/Fiocruz, Rio de Janeiro, Brazil). The tumors were dissociated by mechanical and enzymatic digestion with collagenase (10 mg/mL) in HBSS at 37 °C for 1 h and under agitation. Cells were centrifuged at 200× *g* for 5 min, resuspended in RPMI 1640 supplemented with 10% FBS, 1000 U/mL penicillin and 50 μg/mL streptomycin and maintained at 37 °C in a 5% CO_2_ atmosphere.

### 2.3. Spheroid Growing and Migration Assays

After 5 days of culture, spheroids were treated with different concentrations of doxorubicin (1, 2 and 4 μM) for 72 h. The stock concentration was 10 mM. Cells were retreated at 48 h. DMSO concentration in the medium did not exceed 0.05%. For the migration assay, spheroids were transferred to a 24-well plastic plate without a coverslip and any scaffold before treatment. However, in fluorescence assays, they were transferred to the 24-well plastic plate with a gelatin-coated coverslip. Untreated cells were used as a control. Spheroid growth and cell migration were monitored and photographed daily using phase-contrast microscopy (Nikon Eclipse TS100, Minato-ku, Tokyo, Japan) and the BELView program, version 7.1. Quantification was performed by measuring the diameter or area of MTSs and the diameter of spreading cells over time using the Image J software (https://imagej.net/software/fiji/, accessed on 1 February 2024) (Bethesda, MD, USA).

### 2.4. Necrotic Core Analysis

For evaluation of the necrotic core, after 7 days of culture, spheroids with distinct sizes were incubated for 30 min with Propidium Iodide (PI) (BD Biosciences, San Jose, CA, USA) at a final concentration of 0.5 μg/mL. PI staining was analyzed using the inverted LSM 710 Confocal Laser Scanning Microscope (Zeiss, Oberkochen, Germany). Giemsa staining was also performed to observe the necrotic core of MTSs. Spheroid slices obtained by cryomicrotomy were fixed using Bouin’s fixative solution (picric acid–formalin–acetic acid mixture). The samples were stained in Giemsa solution and dehydrated in an acetone/xylene gradient. Coverslips were mounted with Permount resin, and the images were acquired using bright-field microscopy (Zeiss, Oberkochen, Germany).

### 2.5. Cell Death and Viability Analysis

After 5 days of culture, spheroids were treated with different concentrations of doxorubicin (1, 2 and 4 μM) for 48 or 72 h. Cell death was determined by flow cytometry using the DNA dye 7-amino-actinomycin D (BD Via-Probe™) (BD Bioscience, San Jose, CA, USA). Briefly, after spheroid dissociation with trypsin/EDTA in HBSS without calcium and magnesium, the isolated cells were resuspended in RPMI 1640, washed with cold PBS, and incubated for 20 min at 4 °C with 20 μL of 7-AAD (ready-to-use solution), following the manufacturer’s protocol. Heated cells (60 °C/10 min) were used as a positive control. The samples were acquired with an FACSCalibur flow cytometer (BD Biosciences, San Jose, CA, USA). Data analysis was performed using the software Summit v6.1 (Beckman Coulter, Miami, FL, USA). For cell viability analysis, a minimum of five spheroids/well were incubated with AlamarBlue^®^ reagent (Thermo Scientific, Rockford, IL, USA) for 24 h at 37 °C, according to the manufacturer’s instructions. The absorbance was measured at 570 nm and 600 nm using the microplate spectrophotometer reader Spectramax Plus 384 (Molecular Devices, San Jose, CA, USA). Cell viability was defined by calculating the ratio (in percentage) between each condition treated by the untreated one (control).

### 2.6. Protein Distribution Analysis Using Immunofluorescence and Confocal Microscopy

After 48 h of treatment (4 μM), spheroids were washed twice with ice-cold PBS, stained with methylene blue (for better visualization of spheroids), embedded in Tissue-Tek O.C.T Compound (Sakura Finetek, Chuo-ku, Tokyo, Japan) and frozen in liquid nitrogen until used. The samples were sliced by cryomicrotomy (5 μm thick) and fixed with 4% paraformaldehyde (PFA) for 10 min at 4 °C. After washing, slices were permeabilized with 0.1% Triton X-100. For performing the migration assay, spheroids were fixed/permeabilized with ethanol absolute (Merck, Darmstadt, Germany). Then, non-specific antibody binding was blocked with saline solution (PBS or TBS) containing 4% BSA. The cells were incubated overnight at 4 °C with primary antibodies, including anti-Ki-67, anti-fibronectin, anti-laminin, anti-E-cadherin and anti-vimentin (Table 1). Cells were washed and incubated with the appropriate secondary polyclonal antibodies (Table 1) for 1 h at 37 °C. DNA staining was performed with DAPI 0.2 mg/mL, incubated for 10 min at 20 °C. Slices and migrating spheroids were mounted in ProLong™ Gold Antifade Mountant for further analysis using the inverted confocal microscope described above, and images were processed with the Zen lite 2.3 program (blue edition).

### 2.7. Protein Expression Analysis Using Western Blotting

Cells were washed with PBS twice and scraped with 200 μL of RIPA lysis buffer [50 mM Tris–HCl pH 7.5, 150 mM NaCl, 0.1% SDS, 1% deoxycholate sodium) containing 10% protease inhibitor cocktail (Roche, Indianapolis, IN, USA)] and phosphatase inhibitor cocktail (Sigma Aldrich, St. Louis, MO, USA), and samples were frozen in liquid nitrogen until used. Lysates were sonicated, and protein concentration was determined using the BCA protein quantification kit (Pierce, Rockford, IL, USA). Then, 10 μg of protein was loaded and resolved in 10% SDS-polyacrylamide gels. The proteins were transferred to nitrocellulose membranes (Bio-rad, Hercules, CA, USA) and incubated with 5% skim milk in TBST (TBS and 0.5% Tween 20) for 30 min, followed by incubation with primary antibodies anti-E-cadherin and anti-vimentin, each diluted in TBST, with 5% skim milk overnight at 4 °C (Table 2). Mouse monoclonal anti-GAPDH antibody was used as a loading control (Table 2). Membranes were washed with TBST, followed by incubation with secondary goat anti-rabbit IgG and goat anti-mouse IgG HRP-labeled antibodies (Table 2) for 1 h at 25 °C. Membranes were washed with TBST, incubated with the chemiluminescent kit ECL (Pierce, Rockford, IL, USA) and exposed to X-ray film (Thermo Scientific, Rockford, IL, USA). The densitometry of bands was performed with the software Image Studio Lite 4.0 version. The relative expression of the target proteins (antibodies anti-E-cadherin and anti-vimentin) was determined by the ratio between the values of intensity of its band and the values of the GAPDH band. Relative expression values from treated samples were normalized by the values of untreated cultures at the same time point.

### 2.8. Statistical Analysis

Statistical analyses were performed by GraphPad Prism software 5.0 using one-way ANOVA, two-way ANOVA test with Bonferroni post hoc test, or Mann–Whitney test, as indicated in figure legends. All data were expressed as mean ± SD of at least 3 experiments, analyzing 5 spheroids per experimental condition. For Western blotting analyses, we pooled 60 spheroids per condition [49]. Changes were considered statistically significant when *p* < 0.05 (*), *p* < 0.01 (**) or *p* < 0.001 (***).

## 3. Results

### 3.1. Production and Characterization of the 3D Mammary Spheroid Model Using Cell Line

**Figure 1:**
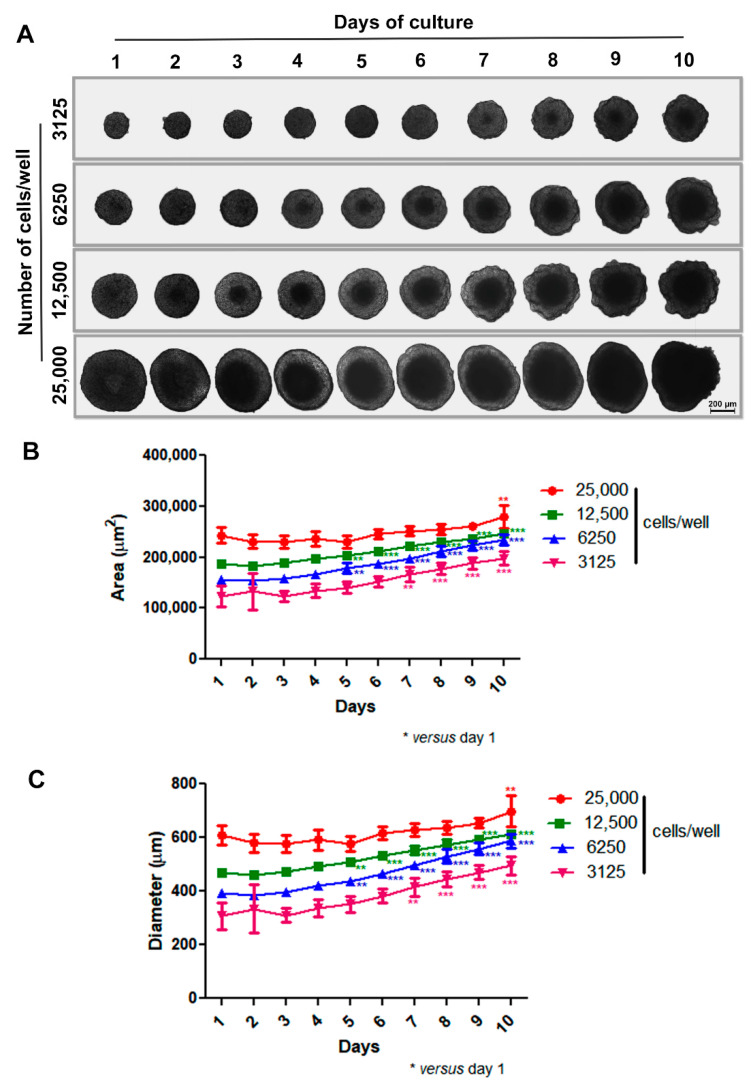
Production of spheroids using MCF-7 cell line. Representative images showing growth and morphology of spheroids with different densities for 10 days by phase-contrast microscopy. Differences in spheroid size were observed depending on the initial number of cells introduced into the wells (**A**). Quantitative data are shown for area and diameter, respectively; all spheroids, except the one with an initial inoculum of 25,000 cells, grew over time, with prominent growth between 5 and 7 days of culture (**B**,**C**). Data are expressed as mean ± SD of one experiment (*n* = 10). ** *p* < 0.01; *** *p* < 0.001. One-way ANOVA test and Bonferroni post hoc test were used.

Our initial step was to develop a scaffold-free 3D model aimed at the spontaneous generation of spheroids without external factors like artificial matrices (e.g., Matrigel). To do this, we coated 96-U-well plastic plates with a thin layer of warm 1% agarose and then treated them for 15–30 min under UV light before use. Based on previous studies by our group [46,47], isolated cells were plated at different densities (3125 to 25,000 cells per well), and spheroid formation and morphology were observed for 10 days using phase-contrast microscopy. In our 3D system, MCF-7 cells adhered to each other and self-assembled, generating a unique spheroid per well in just one day with uniform size. Spheroids of varying sizes exhibited spherical geometry and compact shape, with a progressively more pronounced necrotic core observed over time and concurrent with spheroid enlargement. Notably, the difference in spheroid size was dependent upon the initial number of cells introduced into the wells. Also, all spheroids, except the one with an initial inoculum of 25,000 cells, grew over time, showing prominent growth between 5 and 7 days of culture. The large spheroid phenotype (showing relevant diameter/area increase) was achieved within 10 days (Figure 1A–C).

**Figure 2:**
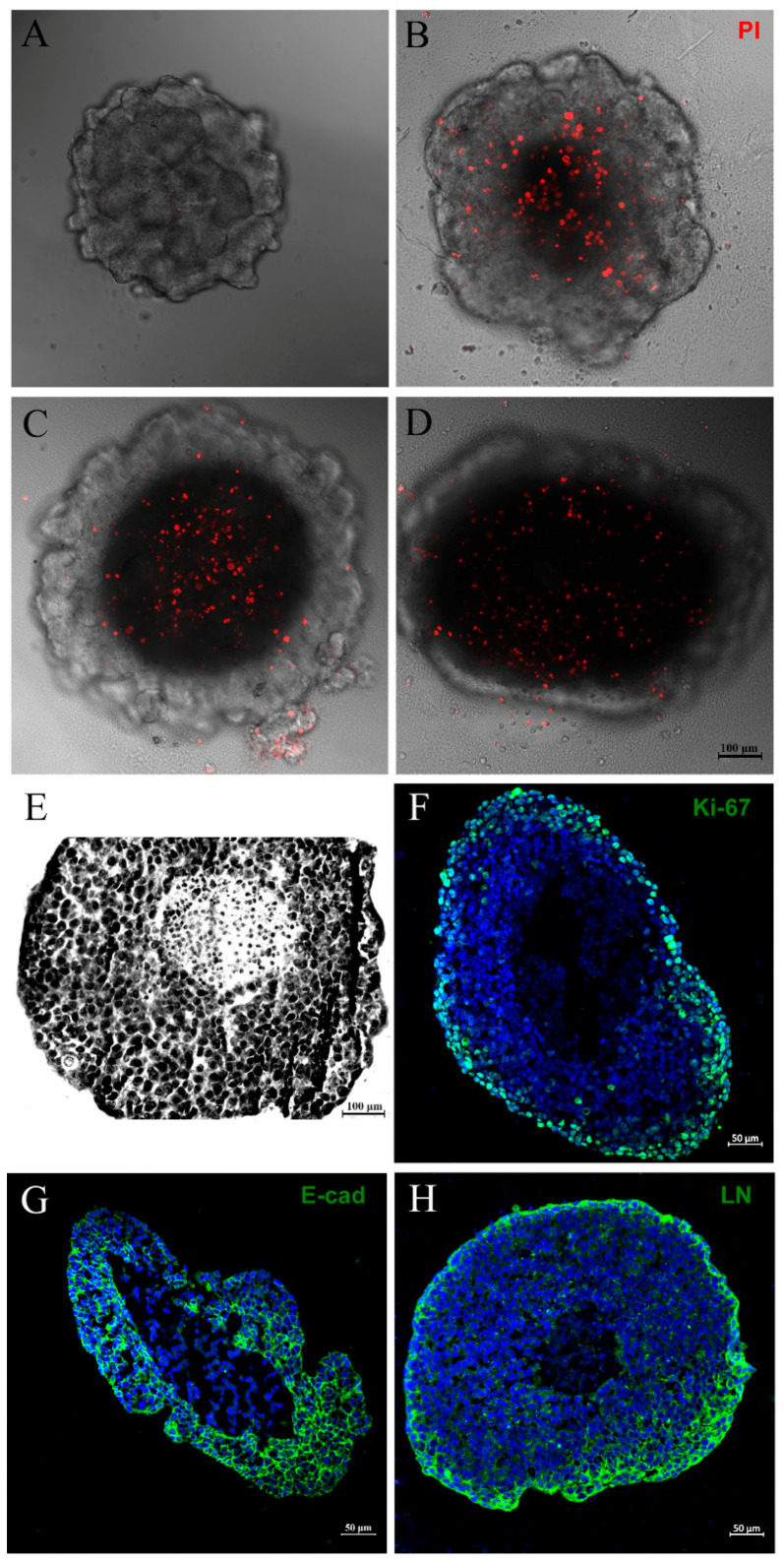
Mammary tumor spheroid mimics characteristics of avascular tumors. PI staining showing a necrotic core in spheroids with distinct densities: 3125 (**A**), 6250 (**B**), 12,500 (**C**) or 25,000 initial cells (**D**). Giemsa staining showing a necrotic center and a large spheroid (6250) (**E**). Immunofluorescence showing Ki-67 (**F**), E-cadherin (E-cad) (**G**), and laminin (LN) (**H**) expression, in green. DNA staining with DAPI can be observed in blue. Ki-67 expression was most prominent at the periphery of the spheroid (**F**). Spheroids presented immunoreactivity for E-cadherin, a well-known epithelial marker, and were able to produce their own matrix, revealed by laminin staining, respectively (**G**,**H**).

Two typical features of large spheroids make them similar to avascular tumors: the necrotic core, as mentioned above, and an outer layer of actively proliferating cells—both originated mainly from poor oxygen, nutrient and growth factor diffusion through the cells [37,38]. Thus, we decided to investigate the necrotic core of spheroids by PI and Giemsa staining, alongside an assessment of cell proliferation status by evaluating Ki-67 expression through immunofluorescence. As expected, we observed PI labeling in the central region of the spheroid, indicating necrosis cell death (Figure 2A–D). The necrotic core could also be seen when spheroids were stained with Giemsa solution (Figure 2E). The Ki-67 expression was most prominent at the spheroid periphery (Figure 2F). These data show that our spheroid model mimics characteristics of avascular tumors: a necrotic center and proliferating cells at the periphery.

For additional characterization, we verified through immunofluorescence that MCF-7 spheroids presented, as expected, immunoreactivity for E-cadherin, a well-known epithelial marker. Cells demonstrated the ability to produce epithelial-like extracellular matrix, evidenced by positive laminin staining (Figure 2G,H).

### 3.2. Production of Spheroids from Mice Mammary Tumors

**Figure 3:**
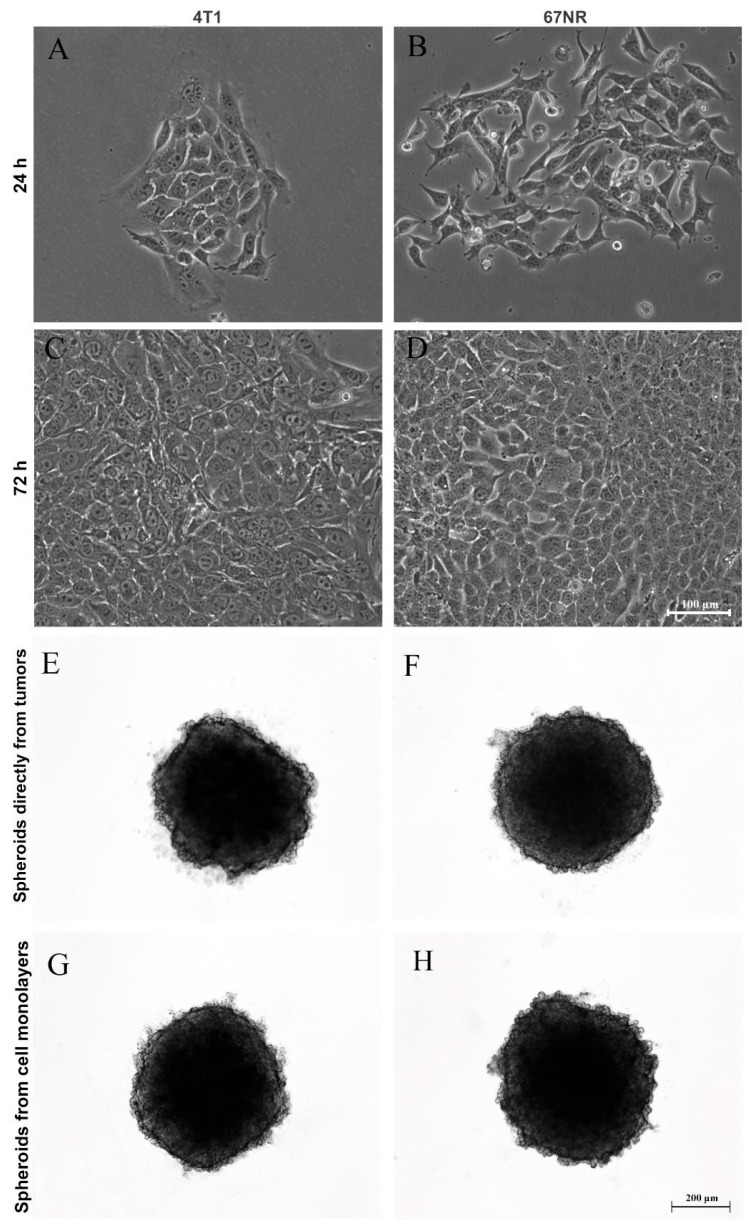
Production of spheroids from mice tumors. Phase-contrast microscopy showing monolayer (2D) culture at 24 h and 72 h (**A**–**D**) and three-dimensional (3D) culture (**E**–**H**) of 4T1 (**A**,**C**,**E**,**G**) and 67NR (**B**,**D**,**F**,**H**) tumor cells. Tumors from both cell lines were dissociated and were capable of adhering in the plastic dishes and growing up as monolayers over time (**A**–**D**), forming spheroids from cells directly isolated from tumors (**E**,**F**) and from cell monolayers (**G**,**H**) after one day of culture.

After generating spheroids from the MCF-7 cell line, we questioned whether it was possible to produce primary mammary spheroids from mice tumors using the spheroid model. Therefore, tumors formed from 4T1 and 67NR cell lines were obtained and dissociated by mechanical and enzymatic digestion with collagenase solution. Importantly, 4T1 tumors are highly metastatic, whereas 67NR tumors are nonmetastatic. Figure 3 shows spheroids produced from cells directly isolated from tumors (E and F) and primary culture in monolayer (G and H). It was not necessary to use a substrate for monolayer cultivation (2D culture), and the cells reached confluence in 72 h (Figure 3A–D). In both cases, spheroid formation occurred after 24 h of cultivation (Figure 3E–H).

### 3.3. Migration Potential of Spheroid Cells In Vitro and Expression of EMT Markers E-Cadherin and Vimentin

**Figure 4:**
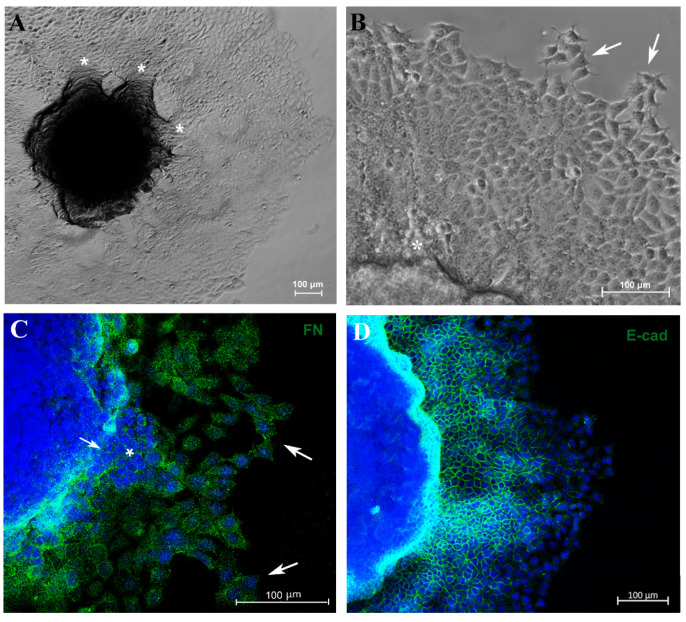

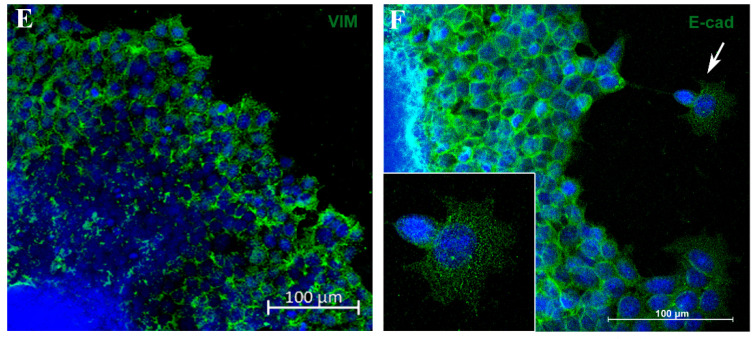
Spheroid migration assay reproduces in vitro features of metastasis. Phase-contrast representative images showing “migration clusters” (asterisk), suggesting collective migration of cells (**A**,**B**). Cells at the periphery of the migration exhibit typical features of motility, such as filopodia (arrow) (**B**). Immunofluorescence staining for fibronectin (FN) (**C**), E-cadherin (E-cad) (**D**,**F**) and vimentin (VIM) (**E**), in green. A “migration cluster” (asterisk) with decreased fibronectin expression, with arrows depicting migrating cells at the periphery with spindle-like morphology (arrow) (**C**). EMT representative process (**D**,**E**); as cells distance from the spheroid, there is a loss of the E-cadherin marker (**D**) and a concurrent acquisition of vimentin (VIM) expression (**E**). Zoom of a cell with flatted-like morphology at the spheroid periphery (inset), a typical feature of mesenchymal cells (**F**). Nuclei were stained with DAPI.

Another important parameter related to tumor progression is the migration/invasion capacity of tumor cells. In an attempt to evaluate these parameters, 5-day culture spheroids were transferred to a 24-well plastic plate to allow cell adhesion. Cells were able to adhere and migrate over time without the use of any scaffold. Furthermore, MCF-7 cells exhibited migration from the spheroid to the plastic surface in distinct directions, forming “migration clusters” that suggest collective migration of cells, a very common property seen in metastatic cancer cells and associated with tumor progression [42] (Figure 4A,B). A decrease in fibronectin expression was observed in sites where tumor cells had extravasated (Figure 4C). Moreover, cells observed at the periphery of the migration zone exhibited typical motility features, such as filopodia and spindle-like morphology (Figure 4A–C).

To verify whether this model reproduces aspects of the metastatic process, such as the EMT, we analyzed E-cadherin (E-cad) and vimentin (VIM) expression by immunofluorescence. Cells close to the MTS border exhibited E-cad labeling, and this marker was reduced as the cells reached the periphery of the migration zone (Figure 4D). In contrast, VIM expression was diminished adjacent to the spheroid border while remaining highly expressed in cells located at the periphery (Figure 4E). Cells with flatted-like or spindle-like morphology, a typical feature of mesenchymal cells, were observed at the peripheric zone (Figure 4C,F). For the first time, aspects of the EMT process in MTSs were reproduced, mimicking in vitro an important feature of metastasis in vivo.

### 3.4. Doxorubicin Induces Cytotoxicity in MTSs

Doxorubicin (dox) is a widely used chemotherapeutic drug used in patients for breast cancer treatment [49]. Our next step was to test the anti-cancer effect of dox on MTSs of the MCF-7 cell line. From this point on, all experiments were conducted using spheroids with an initial density of 6250 cells per well. This choice was made because on the fifth day of culture (also the day chosen to start treatment), spheroids exhibited a necrotic core and proliferating cells at the periphery (Figure 1 and Figure 2), typical features of large spheroids and avascular tumors, as previously mentioned. First, we analyzed the effect of distinct concentrations of dox (1, 2 and 4 μM) on spheroid size in a 72 h time course.

**Figure 5:**
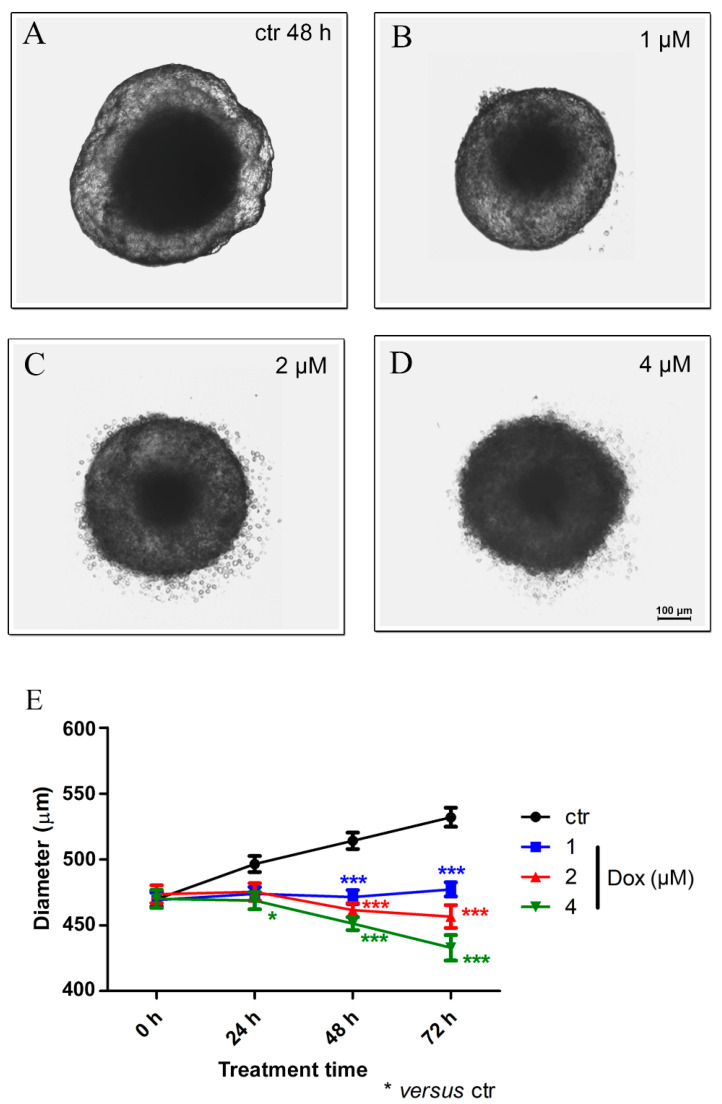
Doxorubicin decreases the spheroid size. Representative images of phase-contrast microscopy of untreated spheroids (ctr) (**A**) and dox treated with 1 μM (**B**), 2 μM (**C**) and 4 μM (**D**) after 48 h of treatment. In addition to a reduction in spheroid size, altered morphology was observed. Graphic representation of spheroid reduction diameter over time along with dox treatment (compared with the untreated control) (**E**). Quantitative data are expressed as the mean ± SD of three experiments, analyzing five spheroids per experimental condition. * *p* < 0.05; *** *p* < 0.001. Two-way ANOVA test and Bonferroni post hoc test were used.

Our results revealed, after 24 h of dox treatment (4 μM), a reduction of 5.7% (*p* < 0.05) in spheroid diameter when compared to the untreated control. A decrease of 8.4% (*p* < 0.001) in spheroid size using 1 μM dox, 10.3% (*p* < 0.001) using 2 μM and 12.2% (*p* < 0.001) with 4 μM was observed after 48 h of treatment. At 72 h, the reduction reached 10.3% (*p* < 0.001) (1 μM), 14.2% (*p* < 0.001) (2 μM) and 18.6% (*p* < 0.001) (4 μM). These results suggest a significant cytotoxic effect of dox in MTSs (Figure 5A–E).

**Figure 6:**
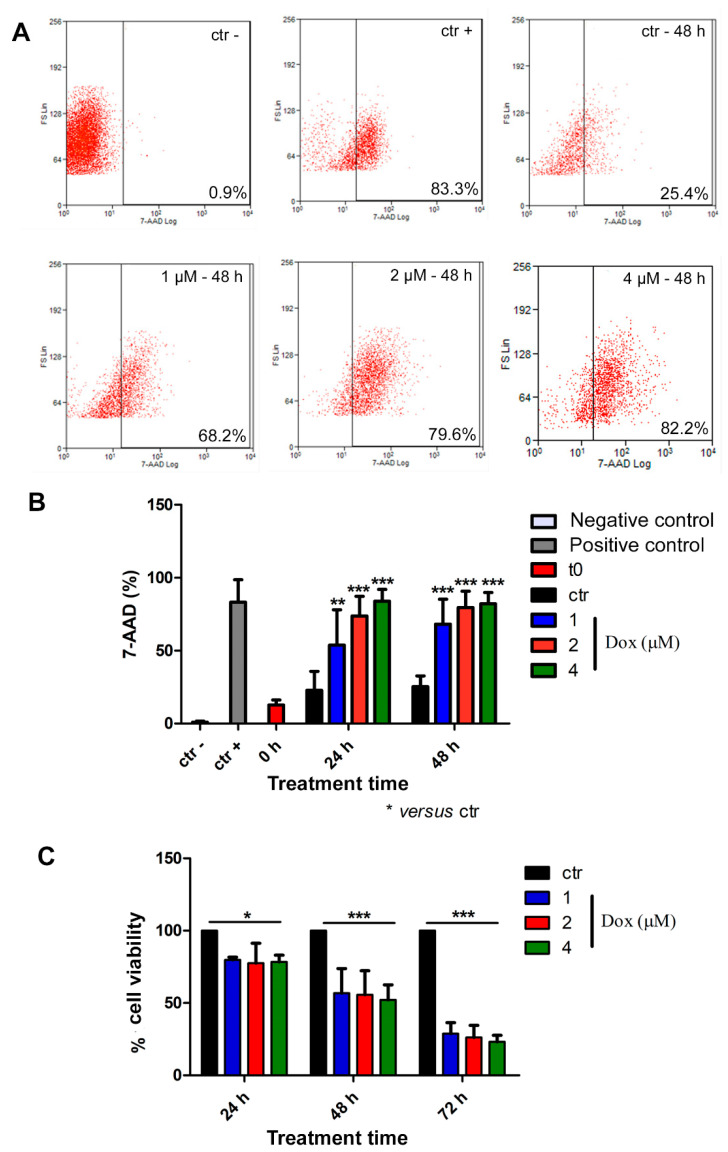
Doxorubicin induces cytotoxicity in spheroids. Representative dot plot of 7-AAD labeling by flow cytometry (**A**). Percentage of 7-AAD-positive cells showed an increase in cell death during dox treatment (**B**). Alamarblue^®^ assay data revealed reduced cell viability during dox treatment at all analyzed time points (**C**). Quantitative data are expressed as the mean ± SD of three experiments, analyzing five spheroids per experimental condition. * *p* < 0.05; ** *p* < 0.01; *** *p* < 0.001. Two-way ANOVA test and Bonferroni post hoc test were used.

Thereafter, we analyzed cell death by flow cytometry. For this purpose, spheroids were dissociated with trypsin, and isolated cells were incubated with the 7-AAD dye. All concentrations of dox induced cell death at all evaluated time points (Figure 6A,B). At 24 h of treatment, the 7-AAD labeling showed a significant increase of 135% (*p* < 0.01), 223% (*p* < 0.001), and 268% (*p* < 0.001) when comparing the control group with the lowest, intermediate, and highest concentrations of doxorubicin, respectively. Following 48 h of treatment, we observed an increase of 169% (*p* < 0.001) in cell death using the lowest dox concentration (1 μM), 214% (*p* < 0.001) using 2 μM and 269% (*p* < 0.001) with 4 μM (Figure 6A,B). We also investigated cell viability by performing Alamarblue^®^ assay, and our results corroborated previous data, indicating a decrease in cell viability during dox treatment at all analyzed time points (Figure 6C). After 24 h of treatment, there was a decrease of 20.3% (*p* < 0.05), 22.4% (*p* < 0.05) and 21.6% (*p* < 0.05) in cell viability when we compared the control condition with 1, 2 and 4 μM concentrations of dox, respectively. After 48 h, we observed a reduction of 43.3% (*p* < 0.001) (1 μM), 44.3% (*p* < 0.001) (2 μM) and 43.6% (*p* < 0.001) (4 μM). Moreover, at 72 h, the effect was even more robust: the decrease reached 71.2% (*p* < 0.001) (1 μM), 73.8% (*p* < 0.001) (2 μM) and 76.8% (*p* < 0.001) (4 μM) (Figure 6C).

### 3.5. Doxorubicin Inhibits Cell Migration in MTSs

**Figure 7:**
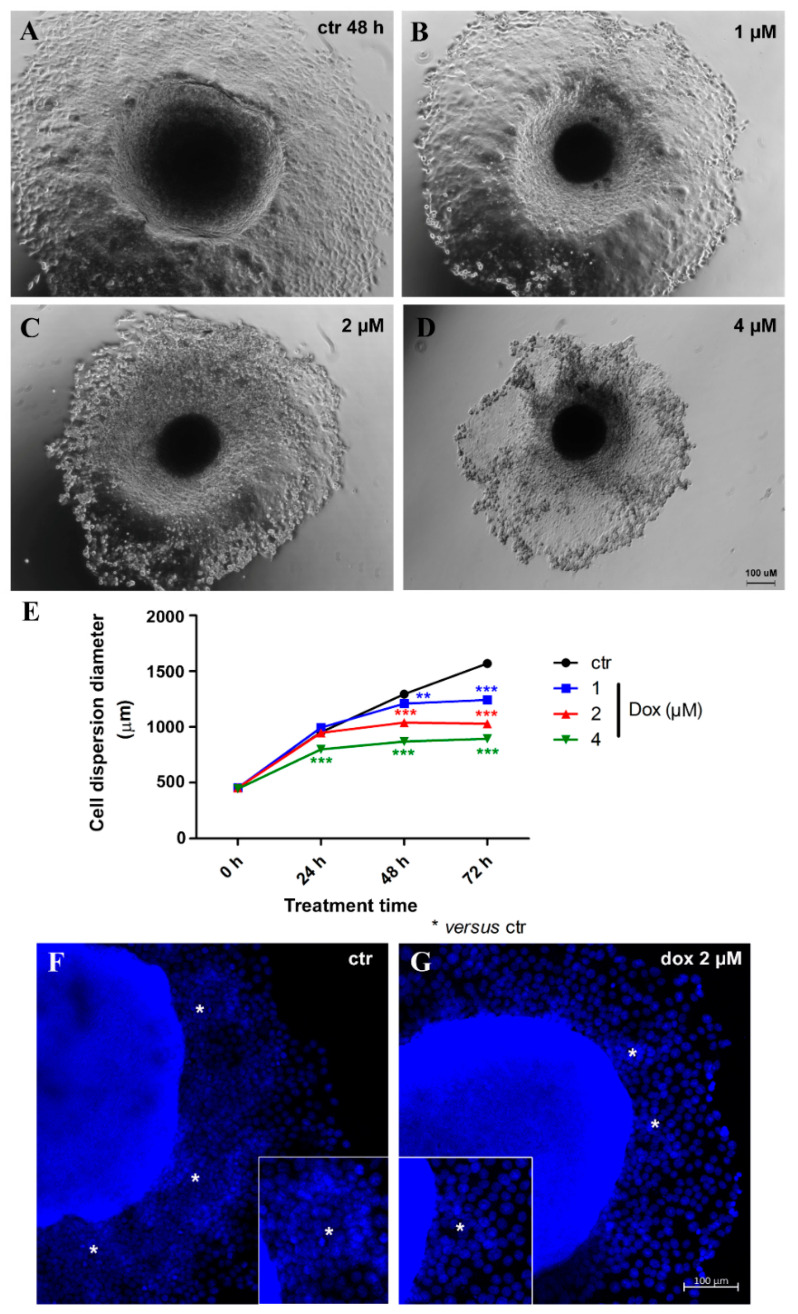
Doxorubicin inhibits cell migration. Representative images of phase-contrast microscopy of untreated spheroids (ctr) (**A**) and treated with 1 μM (**B**), 2 μM (**C**) and 4 μM (**D**) of dox at 48 h of treatment. Graphic showing a decrease in cell spreading from spheroids over time, along with dox treatment, compared with the untreated control (**E**). DNA staining with DAPI in blue, showing a reduction in “migration clusters” (asterisk) (inset) and the number of cells going out of spheroids in the treated condition (**G**) compared with untreated (**F**). Quantitative data are expressed as the mean ± SD of three experiments, analyzing five spheroids per experimental condition. ** *p* < 0.01; *** *p* < 0.001. Two-way ANOVA test and Bonferroni post hoc test were used.

Thereafter, we evaluated the effect of dox treatment on MCF-7 migration/metastatic potential in vitro. Spheroids with 5 days of culture were transferred to a twenty-four-well plastic plate to allow cell adhesion and treated with distinct concentrations of dox (1, 2 and 4 μM) for 72 h. We observed a decrease of 16.1% (*p* < 0.001) in cell spreading from MTSs after 24 h of treatment (4 μM). At 48 h, a decrease of 7.0% (*p* < 0.01) in cell spreading was observed with the lowest concentration of dox, 19.7% (*p* < 0.001) using 2 μM and 32.8% (*p* < 0.001) with 4 μM. A reduction of 20.8% (*p* < 0.001) (1 μM), 34.4% (*p* < 0.001) (2 μM) and 43.0% (*p* < 0.001) (4 μM) was observed after 72 h. These results indicate that dox inhibited the migration of breast tumor cells (Figure 7A,B). Interestingly, migration inhibition by dox was not just observed by a reduction in the diameter of cell spreading—we could also observe a decrease in the “migration clusters” and the number of cells going out of MTSs (Figure 7C).

### 3.6. Doxorubicin Inhibits Aspects of the EMT Process in MTSs

**Figure 8:**
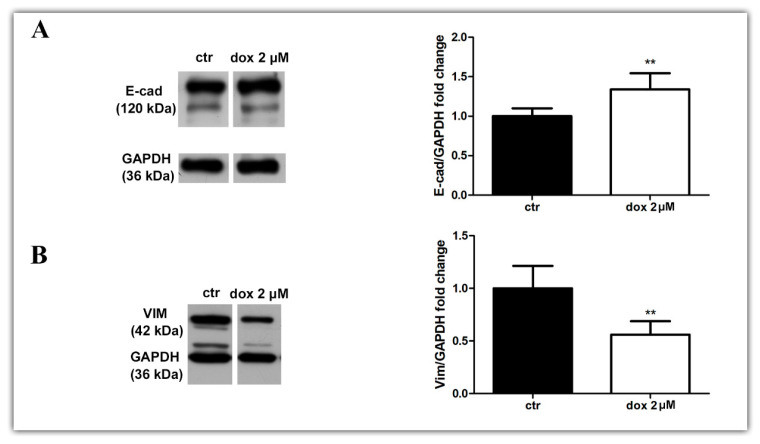

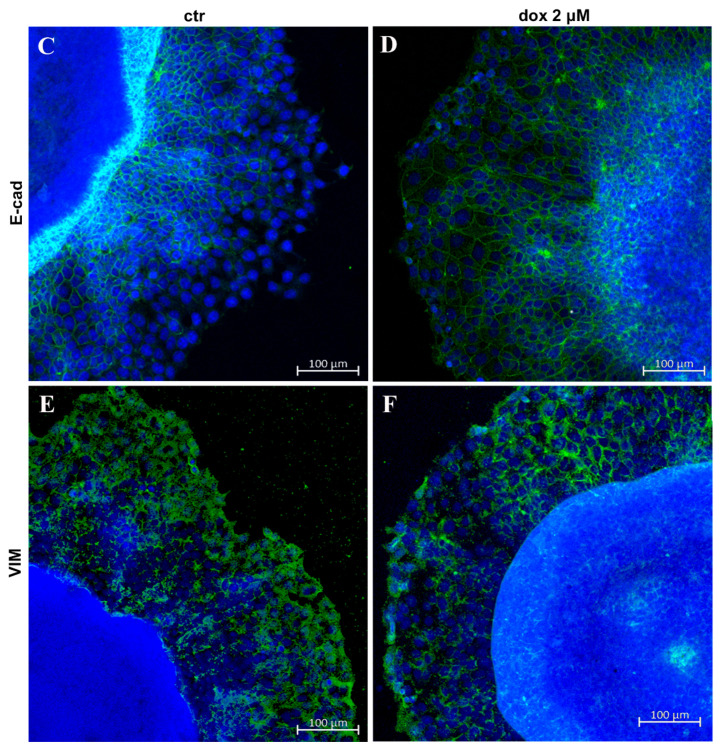
Doxorubicin inhibits aspects of the EMT process. Immunoblotting for evaluation of E-cadherin (E-cad) (**A**) and vimentin (VIM) (**B**) expression during cell migration after 24 h of treatment. Densitometric analyses revealed an increase in E-cad content and a decrease in VIM during dox treatment (**A**,**B**). Immunofluorescence assay showing E-cad staining (**D**) also at the peripheral zone of migration and a decrease in VIM staining (**F**) when cells were treated with dox (**D**,**F**) compared with the untreated condition (**C**,**E**). Nuclei were labeled with DAPI. Quantitative data are expressed as mean ± SD of three experiments. ** *p* < 0.01; Mann–Whitney test.

Finally, we asked whether the dox treatment could influence the EMT process. We analyzed the E-cadherin and vimentin profiles of treated and untreated migrating MTSs by Western blotting and immunofluorescence. Our WB results revealed an increase in E-cad content (33.8%, *p* = 0.0087) and a decrease in VIM content (44.0%, *p* = 0.0043) during dox treatment (Figure 8A,B, Supplementary Figure S1). Interestingly, immunofluorescence showed E-cad staining mainly at cells close to the spheroids in the control condition, while in dox-treated spheroids, this marker was also observed at the peripheral migration zone. We also observed a decrease in VIM expression in this zone (Figure 8C–F). Taken together, these results suggest that, in our 3D culture system, dox inhibited the metastatic potential of breast tumor cells by reducing the collective migration of tumor cells and by inhibiting the EMT process.

## 4. Discussion

Metastasis is a major problem in the treatment of cancer. It is indicative of poor prognosis and has dramatic effects on the survival and quality of life of patients [50,51]. Despite scientific and technological advances that have provided a rise in new markers and therapeutic targets, there is still a need for new therapies that act against metastasis [25,52,53].

Since the 1970s, 3D cell culture systems have contributed to the understanding of cancer biology and therapeutic analysis studies in vitro [54]. This cell culture model better mimics the molecular, morphological and functional features of in vivo tumors [26,31,33], working as a bridge between the traditional in vitro 2D models and in vivo systems [27,29,32], besides reproducing the responses of the patients to chemotherapy drugs [55,56]. The 3D cultures of mammary non-tumor or tumor cells are already well established, and there exist various methods for their production [29,57,58,59,60,61].

There are a variety of in vitro models used to study the migration and invasion of tumor cells. The simplest and most practical are the Boyden chamber and wound-healing methods [40,62,63]. Both can reproduce the migratory/invasive processes but have some limitations. For example, these models fail to picture the EMT process, an important hallmark of metastasis [8,9,11,14,64]. Knowing more about the EMT process makes it possible to design new therapeutic strategies. In 2015, a 3D model of migration was patented that reproduces metastasis features in vivo, such as the expression of EMT markers, through the investigation of completely reversed spheroids in monolayers [42]. Interestingly, microfluidic devices, such as tumor-on-a-chip, have recently been contributing to cancer progression studies [65,66].

Our group has expertise in producing spheroid 3D culture without using an extracellular matrix, including the generation of co-culture systems [44,45,47,48]. Thus, based on these previous studies, we decided to investigate the metastatic potential of cells and the therapeutic response using an MTS model. Our system successfully replicated in vivo metastasis features, like the collective migration of cells and protein expression linked to the epithelial–mesenchymal transition process. Furthermore, we showed that doxorubicin, a well-known chemotherapeutic agent used in the clinic, inhibited this process.

Our first step was to establish a 3D model without using external factors, like mechanical forces and growing factors provided by artificial matrices, that could influence spheroid formation. Here, we observed that the cells adhered to each other spontaneously and formed compact spheroids in just one day of culture, regardless of the initial number of cells introduced into the wells. In addition, they grew according to the time of culture, except for the sample generated from a substantial initial cell inoculum characterized by a large-onset spheroid phenotype. This deviation may be attributed to the extensive hypoxia induced within it, disrupting its normal growth. Importantly, generating a unique spheroid per well with uniform size favors reproducible results and makes this system an important tool for high-throughput screening. Indeed, spheroids produced using the liquid overlay technique on inert surfaces (the same model used in this study) are recognized as the best model for this type of test for the reasons mentioned above [38,39,67,68].

Then, as a complementary characterization of the model, we also evaluated proteins expressed by cells of epithelial origin, such as E-cadherin, an adherens junction protein, and laminin, an extracellular matrix (EM)/basal lamina protein, through immunofluorescence. The MTSs exhibited characteristic labeling for E-cadherin and were able to produce their own EM (laminin). Some studies have already shown that mammary non-tumors and tumor spheroids express cell junction proteins, such as E-cadherin and ZO-1, in addition to integrins, in a similar way as happens in vivo [33,69]. Interestingly, changes in some of these components have been related to loss of cell polarity and tumor development [33,70,71]. The secretion of laminin and other components of EM, such as fibronectin, collagen IV and tenascin, has already been evidenced in mammary spheroids produced from both non-tumor and tumor cells [34,72,73] and even in co-cultivation systems with endothelial cells and fibroblasts [74]. The cell’s ability to produce its own EM is critical to the development of a functional phenotype and portrays much more reliably the way it occurs in vivo [34,75].

Avascular tumors, when they reach between 0,5 and 1 mm3 or micrometastasis, are characterized by having a necrotic core with quiescent cells and a peripheric zone with cells that are actively proliferative, and they originate mainly due to poor oxygen, nutrient, metabolite, and growth factor diffusion through the cells. Such features can be achieved by large MTSs (between 200 μm and 500 μm in diameter) [27,35,76]. In our 3D system results, the spheroids showed a necrotic core, evidenced by PI labeling. We also observed active proliferating cells at the periphery zone through Ki-67 staining. Cells at the periphery are in intimate contact with the medium, consuming more nutrients and oxygen, whereas the central counterpart has insufficient oxygen and nutrient perfusion, resulting in non-proliferating, necrotic and hypoxic cells [35,36]. The shared heterogeneity observed in avascular tumors, micrometastasis and large spheroids renders them relevant models for pathophysiological studies.

Here, we also demonstrated that besides the generation of spheroids from the MCF-7 cell line, our spheroid model supports the establishment of primary mammary spheroids from mice tumors induced from cell lines with distinct invasive capacities (4T1 and 67NR). Using enzymatic protocols previously used for the dissociation of tumors from patients [77], we confirmed the dissociation of cells from tumors and observed their growth during the culture period either as a monolayer or in the 3D system, forming compact spheroids in just one day, as with the MCF-7 cell line. Although there is a lack of studies using this approach, it could be further explored to enhance our understanding of cancer biology and the role of the microenvironment on tumor progression, as well as to provide insights about new therapeutic strategies. Importantly, a work showed that breast cancer spheroids originating from patient-derived xenografts (PDXs) exhibited similar Ki-67 and caspase-3 expression when compared with the patient’s original tumor and fresh PDX tumor than traditional 2D culture [78], showing again that 3D culture better recapitulates the tumor characteristics of experimental models and from patients. Indeed, in the past few years, studies have shown that mammary tumor spheroids respond to in vitro treatment in a similar way to their original tumors when treated with the same chemotherapy drug used by patients or current guideline treatment recommendations for breast cancer [55,56]. Thus, it is considered a promising model for the evaluation of personalized therapy [55,56,79].

Regarding the migration assay in an adherent surface, our results showed that cells adhered and migrated over culture time in different directions, forming “migration clusters” suggestive of collective migration, a very common type of migration in breast cancer [18], which is characterized by the preservation of functional cell–cell junctions like E-cad.

An in vivo study using spheroids from 4T1 and MMT (murine mammary tumor cells, E-cad-negative) implanted into the murine mammary fat demonstrated that cells left the spheroid and migrated collectively, and some of them presented spindle-like or elongated morphology; cells were able to detach and migrate individually (suggesting an EMT process), forming micro- and macrometastases in the lung [17]. Here, for the first time, we demonstrated that the migration model reproduced aspects of the EMT process in MTSs, confirmed by the reduction in the E-cad marker and the increase in VIM protein as the cells moved farther away from the spheroid. Cells at the periphery exhibited a flatted-like or spindle-like shape, typical characteristics of mesenchymal cells. In this study, besides recapitulating the features of tumors in vivo, we mimicked the migration of cells out of small cancer clusters that occur in vivo and reproduced the EMT process [8,9,80,81].

Doxorubicin is a widely used chemotherapeutic drug used in the clinic for the treatment of a variety of cancers, such as breast cancer [49]. The main mechanisms of action of dox include DNA intercalation, topoisomerase II inhibition, and free radical formation inside cells [82]. In an attempt to validate our 3D culture system, we investigated the dox effect on MTSs. As expected, we demonstrated that dox induced cytotoxicity in MTSs, reducing spheroid diameter and cell viability and inducing cell death. Some data from the literature indicate that dox exhibits cytotoxic effects in 3D spheroids; however, this effect is less pronounced when compared with cells growing in a monolayer and treated with the same drug concentration [83,84,85,86,87], possibly because spheroids are more resistant to treatment. Interestingly, the complex organization of ECM proteins, as well as the lower proliferation rate of spheroid cells, has been implicated in promoting the resistance of mammary cancer cells to dox in a 3D culture model using scaffold matrices [88]. Furthermore, studies have shown that tumor spheroids express genes related to drug resistance, such as MDR1 [89], and harbor a significant population of cancer stem cells, which are characterized by a higher metastatic potential and are well-documented for their increased resistance to therapy [42,90,91]. The characteristic of 3D models being more resistant to drugs does not reduce its importance or applicability for therapeutic tests; on the contrary, by better mimicking features of in vivo systems, such as the therapeutic resistance found in solid tumors, it makes the results much more reproducible and reliable [89].

Although it has excellent anti-tumor activity, dox induces serious side effects, such as immunosuppression and cardiotoxicity [92,93]. Beyond the side effects, the prolonged use of dox has also been linked to drug resistance, both factors being the principal challenges for treatment effectiveness [94,95]. In this regard, new formulations or even combinations of dox with other drugs that aim to reduce its concentration are effective strategies to mitigate side effects [96]. However, reducing the drug concentration may lead to the development of resistance and the induction of metastatic potential [97,98]. Low dox concentrations have been implicated in favoring the metastatic process of triple-negative breast cancer cells through activation of TGF-β1 [99] and upregulation of the RhoA/MLC [100] or Twist1 [101] pathway.

We also investigated the dox treatment effect on the migration and metastatic potential of MCF-7 cells. Our results demonstrated that dox reduced cell propagation from the spheroids. Moreover, we observed that dox not only inhibited cell migration but also interfered with the metastatic potential of mammary tumor cells, reducing collective migration and the EMT process. Inhibition of the EMT process was observed by expressive vimentin reduction and E-cadherin increase. Through the immunofluorescence approach, it was possible to examine this phenomenon in greater detail, depicting the E-cad label in the peripheral zone of migration and the decrease in VIM expression in that same region.

Discrepancies between our findings and the aforementioned literature data may arise from the fact that these studies were conducted using very low doses of doxorubicin (maximum of 800 nM). A work using a different chemotherapeutic agent, paclitaxel, demonstrated that low doses (1 mg/kg) of this drug favored mammary tumor cell metastasis to the liver in a murine model, while high doses (20 mg/kg) reduced this process [102]. Another bias could be the lack of an ideal in vitro model to study the metastatic potential of cells. In 2015, a spheroid migration assay similar to ours was patented. They showed that completely reversed spheroids reproduce metastasis features in vivo, such as migration/invasion, chemoresistance and cancer stem cell marker expression (CD133, CD90, EpCAM) [42]. Differently, rather than investigate spheroids that had completely reversed to a monolayer, we aimed to characterize metastasis features during the reversal process. Transposing into an in vivo situation, we believed it would be more effective to study and intervene in the metastasis process at its initiation rather than after it had already been established.

## 5. Conclusions

In conclusion, our results contribute to advances in the field of precision medicine in cancer using a spheroid 3D culture system to evaluate therapeutic response using doxorubicin, a widely used chemotherapeutic agent in the clinic. Our migration assay reproduced features of metastasis in vivo, thus becoming an essential tool for studying cancer progression in vitro and testing antimetastatic agents. Furthermore, we showed that doxorubicin inhibited these processes, suggesting a role of this drug in the inhibition of the metastatic process, which should be further explored.

## Figures and Tables

**Table 1:** Antibodies used in immunofluorescence assays.

Antibody	Manufacturer	Catalog Number	Antibody Dilution
Anti-E-cadherin antibody, rabbit monoclonal	Sigma-Aldrich	SAB5500022	1:100
Anti-vimentin antibody, mouse monoclonal	Sigma-Aldrich	V5255	1:200
Anti-fibronectin antibody, rabbit polyclonal	Sigma-Aldrich	F3648	1:200
Anti-laminin antibody, rabbit polyclonal	Sigma-Aldrich	L9393	1:50
Anti-Ki-67 antibody, rabbit recombinant monoclonal	Abcam	ab16667	1:100
Donkey anti-mouse IgG secondary antibody (Alexa Fluor™ 594)	Thermo Scientific	A21203	1:800
Donkey anti-rabbit IgG secondary antibody (Alexa Fluor™ 488)	Thermo Scientific	A21206	1:800

**Table 2:** Antibodies used in Western blotting assays.

Antibody	Manufacturer	Catalog Number	Antibody Dilution
Anti-E-cadherin antibody, rabbit monoclonal	Sigma-Aldrich	SAB5500022	1:2000
Anti-vimentin antibody, mouse monoclonal	Sigma-Aldrich	V5255	1:3000
Anti-GAPDH antibody, mouse monoclonal	Fitzgerald	10R-G109a	1:80,000
Goat anti-mouse IgG secondary antibody (HRP)	R&D Systems	HAF007	1:1000
Goat anti-rabbit IgG secondary antibody (HRP)	R&D Systems	HAF008	1:1000

## Data Availability

The datasets of the current study are available from the corresponding author upon reasonable request.

## Supplementary Materials

The following supporting information can be downloaded at: https://www.mdpi.com/article/10.3390/biology13070463/s1, Figure S1: Figure shows Western blotting in triplicate for E-cad (A) and VIM (B), related to Figure 8.

## References

[1] WHO (2024). Breast Cancer. https://www.who.int/news-room/fact-sheets/detail/breast-cancer.

[2] Cecilio A.P., Takakura E.T., Jumes J.J., Dos Santos J.W., Herrera A.C., Victorino V.J., Panis C. (2015). Breast cancer in Brazil: Epidemiology and treatment challenges. Breast Cancer.

[3] (2023). BCNA. https://www.bcna.org.au/resource-hub/articles/types-of-breast-cancer/.

[4] Lee B.L., Liedke P.E.R., Barrios C.H., Simon S.D., Finkelstein D.M., Goss P.E. (2012). Breast cancer in Brazil: Present status and future goals. Lancet Oncol..

[5] Hanahan D., Weinberg R.A. (2000). The hallmarks of cancer. Cell.

[6] Hanahan D., Weinberg R.A. (2011). Hallmarks of cancer: The next generation. Cell.

[7] Hanahan D. (2022). Hallmarks of Cancer: New Dimensions. Cancer Discov..

[8] Drasin D.J., Robin T.P., Ford H.L. (2011). Breast cancer epithelial-to-mesenchymal transition: Examining the functional consequences of plasticity. Breast Cancer Res..

[9] Heerboth S., Housman G., Leary M., Longacre M., Byler S., Lapinska K., Willbanks A., Sarkar S. (2015). EMT and tumor metastasis. Clin. Transl. Med..

[10] Kang E., Seo J., Yoon H., Cho S. (2021). The Post-Translational Regulation of Epithelial-Mesenchymal Transition-Inducing Transcription Factors in Cancer Metastasis. Int. J. Mol. Sci..

[11] Seyfried T.N., Huysentruyt L.C. (2013). On the origin of cancer metastasis. Crit. Rev. Oncog..

[12] Moreno-Bueno G., Portillo F., Cano A. (2008). Transcriptional regulation of cell polarity in EMT and cancer. Oncogene.

[13] Zhitnyak I.Y., Rubtsova S.N., Litovka N.I., Gloushankova N.A. (2020). Early Events in Actin Cytoskeleton Dynamics and E-Cadherin-Mediated Cell-Cell Adhesion during Epithelial-Mesenchymal Transition. Cells.

[14] Bakir B., Chiarella A.M., Pitarresi J.R., Rustgi A.K. (2020). EMT, MET, Plasticity, and Tumor Metastasis. Trends Cell Biol..

[15] Satelli A., Li S. (2011). Vimentin in cancer and its potential as a molecular target for cancer therapy. Cell Mol. Life Sci..

[16] Gao Y., Wang Z., Hao Q., Li W., Xu Y., Zhang J., Zhang W., Wang S., Liu S., Li M. (2017). Loss of ERα induces amoeboid-like migration of breast cancer cells by downregulating vinculin. Nat. Commun..

[17] Ilina O., Campanello L., Gritsenko P.G., Vullings M., Wang C., Bult P., Losert W., Friedl P. (2018). Intravital microscopy of collective invasion plasticity in breast cancer. Dis. Model. Mech..

[18] Khalil A.A., Ilina O., Gritsenko P.G., Bult P., Span P.N., Friedl P. (2017). Collective invasion in ductal and lobular breast cancer associates with distant metastasis. Clin. Exp. Metastasis.

[19] Madsen C.D., Sahai E. (2010). Cancer Dissemination-Lessons from Leukocytes. Dev. Cell.

[20] de Abreu F.B., Schwartz G.N., Wells W.A., Tsongalis G.J. (2014). Personalized therapy for breast cancer. Clin. Genet..

[21] Dumbrava E.I., Meric-Bernstam F. (2018). Personalized cancer therapy- leveraging a knowledge base for clinical decision-making. Cold Spring Harb. Mol. Case Stud..

[22] Higgins M.J., Baselga J. (2011). Targeted therapies for breast cancer. J. Clin. Investig..

[23] Masoud V., Pages G. (2017). Targeted therapies in breast cancer: New challenges to fight against resistance. World J. Clin. Oncol..

[24] Mendes D., Alves C., Afonso N., Cardoso F., Passos-Coelho J.L., Costa L., Andrade S., Batel-Marques F. (2015). The benefit of HER2-targeted therapies on overall survival of patients with metastatic HER2-positive breast cancer—A systematic review. Breast Cancer Res..

[25] Ganesh K., Massague J. (2021). Targeting metastatic cancer. Nat. Med..

[26] Chaicharoenaudomrung N., Kunhorm P., Noisa P. (2019). Three-dimensional cell culture systems as an in vitro platform for cancer and stem cell modeling. World J. Stem Cells.

[27] Ham S.L., Joshi R., Thakuri P.S., Tavana H. (2016). Liquid-based three-dimensional tumor models for cancer research and drug discovery. Exp. Biol. Med..

[28] Urzì O., Gasparro R., Costanzo E., De Luca A., Giavaresi G., Fontana S., Alessandro R. (2023). Three-Dimensional Cell Cultures: The Bridge between In Vitro and In Vivo Models. Int. J. Mol. Sci..

[29] Weigelt B., Bissell M.J. (2008). Unraveling the microenvironmental influences on the normal mammary gland and breast cancer. Semin. Cancer Biol..

[30] Friedrich J., Seidel C., Ebner R., Kunz-Schughart L.A. (2009). Spheroid-based drug screen: Considerations and practical approach. Nat. Protoc..

[31] Goliwas K.F., Richter J.R., Pruitt H.C., Araysi L.M., Anderson N.R., Samant R.S., Lobo-Ruppert S.M., Berry J.L., Frost A.R. (2017). Methods to Evaluate Cell Growth, Viability, and Response to Treatment in a Tissue Engineered Breast Cancer Model. Sci. Rep..

[32] Singha B., Laski J., Valdés Y.R., Liu E., Dimattia G.E., Shepherd T.G. (2020). Inhibiting ULK1 kinase decreases autophagy and cell viability in high-grade serous ovarian cancer spheroids. Am. J. Cancer Res..

[33] Vidi P.A., Bissell M.J., Lelievre S.A. (2013). Three-dimensional culture of human breast epithelial cells: The how and the why. Methods Mol. Biol..

[34] Timmins N.E., Harding F.J., Smart C., Brown M.A., Nielsen L.K. (2005). Method for the generation and cultivation of functional three-dimensional mammary constructs without exogenous extracellular matrix. Cell Tissue Res..

[35] Groebe K., Mueller-Klieser W. (1991). Distributions of oxygen, nutrient, and metabolic waste concentrations in multicellular spheroids and their dependence on spheroid parameters. Eur. Biophys. J..

[36] Mehta G., Hsiao A.Y., Ingram M., Luker G.D., Takayama S. (2012). Opportunities and challenges for use of tumor spheroids as models to test drug delivery and efficacy. J. Control Release.

[37] LaBarbera D.V., Reid B.G., Yoo B.H. (2012). The multicellular tumor spheroid model for high-throughput cancer drug discovery. Expert. Opin. Drug Discov..

[38] Utama R.H., Atapattu L., O’Mahony A.P., Fife C.M., Baek J., Allard T., O’Mahony K.J., Ribeiro J.C.C., Gaus K., Kavallaris M. (2020). A 3D Bioprinter Specifically Designed for the High-Throughput Production of Matrix-Embedded Multicellular Spheroids. IScience.

[39] Yang L., Pijuan-Galito S., Rho H.S., Vasilevich A.S., Eren A.D., Ge L., Habibović P., Alexander M.R., de Boer J., Carlier A. (2021). High-Throughput Methods in the Discovery and Study of Biomaterials and Materiobiology. Chem. Rev..

[40] Kramer N., Walzl A., Unger C., Rosner M., Krupitza G., Hengstschlager M., Dolznig H. (2013). In vitro cell migration and invasion assays. Mutat. Res..

[41] Langthasa J., Sarkar P., Narayanan S., Bhagat R., Vadaparty A., Bhat R. (2021). Extracellular matrix mediates moruloid-blastuloid morphodynamics in malignant ovarian spheroids. Life Sci. Alliance.

[42] Kunjithapatham R., Karthikeyan S., Geschwind J.F., Kieserman E., Lin M., Fu D.X., Ganapathy-Kanniappan S. (2014). Reversal of anchorage-independent multicellular spheroid into a monolayer mimics a metastatic model. Sci. Rep..

[43] de Barros A.P., Takiya C.M., Garzoni L.R., Leal-Ferreira M.L., Dutra H.S., Chiarini L.B., Meirelles M.N., Borojevic R., Rossi M.I. (2010). Osteoblasts and bone marrow mesenchymal stromal cells control hematopoietic stem cell migration and proliferation in 3D in vitro model. PLoS ONE.

[44] Garzoni L.R., Adesse D., Soares M.J., Rossi M.I., Borojevic R., de Meirelles Mde N. (2008). Fibrosis and hypertrophy induced by Trypanosoma cruzi in a three-dimensional cardiomyocyte-culture system. J. Infect. Dis..

[45] Garzoni L.R., Rossi M.I., de Barros A.P., Guarani V., Keramidas M., Balottin L.B., Adesse D., Takiya C.M., Manso P.P., Otazu I.B. (2009). Dissecting coronary angiogenesis: 3D co-culture of cardiomyocytes with endothelial or mesenchymal cells. Exp. Cell Res..

[46] Rossi M.I., Barros A.P., Baptista L.S., Garzoni L.R., Meirelles M.N., Takiya C.M., Pascarelli B.M., Dutra H.S., Borojevic R. (2005). Multicellular spheroids of bone marrow stromal cells: A three-dimensional in vitro culture system for the study of hematopoietic cell migration. Braz. J. Med. Biol. Res..

[47] Ferrão P.M., Nisimura L.M., Moreira O.C., Land M.G., Pereira M.C., de Mendonça-Lima L., Araujo-Jorge T.C., Waghabi M.C., Garzoni L.R. (2018). Inhibition of TGF-β pathway reverts extracellular matrix remodeling in *T. cruzi*-infected cardiac spheroids. Exp. Cell Res..

[48] Nisimura L.M., Ferrão P.M., Nogueira A.D.R., Waghabi M.C., Meuser-Batista M., Moreira O.C., Urbina J.A., Garzoni L.R. (2020). Effect of Posaconazole in an in vitro model of cardiac fibrosis induced by *Trypanosoma cruzi*. Mol. Biochem. Parasitol..

[49] Hernandez-Aya L.F., Gonzalez-Angulo A.M. (2013). Adjuvant systemic therapies in breast cancer. Surg. Clin. N. Am..

[50] Al-Mahmood S., Sapiezynski J., Garbuzenko O.B., Minko T. (2018). Metastatic and triple-negative breast cancer: Challenges and treatment options. Drug Deliv. Transl. Res..

[51] Parker A.L., Benguigui M., Fornetti J., Goddard E., Lucotti S., Insua-Rodríguez J., Wiegmans A.P., Early Career Leadership Council of the Metastasis Research Society (2022). Current challenges in metastasis research future innovation for clinical translation. Clin. Exp. Metastasis.

[52] Qian C.N., Mei Y., Zhang J. (2017). Cancer metastasis: Issues and challenges. Chin. J. Cancer.

[53] Steeg P.S. (2016). Targeting metastasis. Nat. Rev. Cancer.

[54] Emerman J.T., Enami J., Pitelka D.R., Nandi S. (1977). Hormonal effects on intracellular and secreted casein in cultures of mouse mammary epithelial cells on floating collagen membranes. Proc. Natl. Acad. Sci. USA.

[55] Halfter K., Ditsch N., Kolberg H.C., Fischer H., Hauzenberger T., von Koch F.E., Bauerfeind I., von Minckwitz G., Funke I., Crispin A. (2015). Prospective cohort study using the breast cancer spheroid model as a predictor for response to neoadjuvant therapy—The SpheroNEO study. BMC Cancer.

[56] Halfter K., Hoffmann O., Ditsch N., Ahne M., Arnold F., Paepke S., Grab D., Bauerfeind I., Mayer B. (2016). Testing chemotherapy efficacy in HER2 negative breast cancer using patient-derived spheroids. J. Transl. Med..

[57] Hong S., Song J.M. (2022). 3D bioprinted drug-resistant breast cancer spheroids for quantitative in situ evaluation of drug resistance. Acta Biomater..

[58] Bahcecioglu G., Basara G., Ellis B.W., Ren X., Zorlutuna P. (2020). Breast cancer models: Engineering the tumor microenvironment. Acta Biomater..

[59] Carter E.P., Gopsill J.A., Gomm J.J., Jones J.L., Grose R.P. (2017). A 3D in vitro model of the human breast duct: A method to unravel myoepithelial-luminal interactions in the progression of breast cancer. Breast Cancer Res..

[60] Nath S., Devi G.R. (2016). Three-dimensional culture systems in cancer research: Focus on tumor spheroid model. Pharmacol. Ther..

[61] Weiswald L.B., Bellet D., Dangles-Marie V. (2015). Spherical cancer models in tumor biology. Neoplasia.

[62] Huang Z., Yu P., Tang J. (2020). Characterization of Triple-Negative Breast Cancer MDA-MB-231 Cell Spheroid Model. Onco Targets Ther..

[63] Liang C.C., Park A.Y., Guan J.L. (2007). In vitro scratch assay: A convenient and inexpensive method for analysis of cell migration in vitro. Nat. Protoc..

[64] Guan X. (2015). Cancer metastases: Challenges and opportunities. Acta Pharm. Sin. B.

[65] Bouchalova P., Bouchal P. (2022). Current methods for studying metastatic potential of tumor cells. Cancer Cell Int..

[66] Wang Y., Qin J. (2023). Advances in human organoids-on-chips in biomedical research. Life Med..

[67] Kunz-Schughart L.A., Freyer J.P., Hofstaedter F., Ebner R. (2004). The use of 3-D cultures for high-throughput screening: The multicellular spheroid model. J. Biomol. Screen..

[68] Lv D., Hu Z., Lu L., Lu H., Xu X. (2017). Three-dimensional cell culture: A powerful tool in tumor research and drug discovery. Oncol. Lett..

[69] Wang F., Hansen R.K., Radisky D., Yoneda T., Barcellos-Hoff M.H., Petersen O.W., Turley E.A., Bissell M.J. (2002). Phenotypic reversion or death of cancer cells by altering signaling pathways in three-dimensional contexts. J. Natl. Cancer Inst..

[70] Chandramouly G., Abad P.C., Knowles D.W., Lelièvre S.A. (2007). The control of tissue architecture over nuclear organization is crucial for epithelial cell fate. J. Cell Sci..

[71] Halaoui R., Rejon C., Chatterjee S.J., Szymborski J., Meterissian S., Muller W.J., Omeroglu A., McCaffrey L. (2017). Progressive polarity loss and luminal collapse disrupt tissue organization in carcinoma. Genes. Dev..

[72] Park H., Helfman D.M. (2019). Up-regulated fibronectin in 3D culture facilitates spreading of triple negative breast cancer cells on 2D through integrin β-5 and Src. Sci. Rep..

[73] Streuli C.H., Bailey N., Bissell M.J. (1991). Control of mammary epithelial differentiation: Basement membrane induces tissue-specific gene expression in the absence of cell-cell interaction and morphological polarity. J. Cell Biol..

[74] Correa de Sampaio P., Auslaender D., Krubasik D., Failla A.V., Skepper J.N., Murphy G., English W.R. (2012). A heterogeneous in vitro three dimensional model of tumour-stroma interactions regulating sprouting angiogenesis. PLoS ONE.

[75] Bissell M.J., Bilder D. (2003). Polarity determination in breast tissue: Desmosomal adhesion, myoepithelial cells, and laminin 1. Breast Cancer Res..

[76] Kunz-Schughart L.A., Kreutz M., Knuechel R. (1998). Multicellular spheroids: A three-dimensional in vitro culture system to study tumour biology. Int. J. Exp. Pathol..

[77] Vázquez S.M., Mladovan A., Garbovesky C., Baldi A., Lüthy I.A. (2004). Three novel hormone-responsive cell lines derived from primary human breast carcinomas: Functional characterization. J. Cell Physiol..

[78] Imamura Y., Mukohara T., Shimono Y., Funakoshi Y., Chayahara N., Toyoda M., Kiyota N., Takao S., Kono S., Nakatsura T. (2015). Comparison of 2D- and 3D-culture models as drug-testing platforms in breast cancer. Oncol. Rep..

[79] Halfter K., Mayer B. (2017). Bringing 3D tumor models to the clinic—Predictive value for personalized medicine. Biotechnol. J..

[80] Kalluri R., Weinberg R.A. (2009). The basics of epithelial-mesenchymal transition. J. Clin. Investig..

[81] Zhang Y., Weinberg R.A. (2018). Epithelial-to-mesenchymal transition in cancer: Complexity and opportunities. Front. Med..

[82] Tacar O., Sriamornsak P., Dass C.R. (2013). Doxorubicin: An update on anticancer molecular action, toxicity and novel drug delivery systems. J. Pharm. Pharmacol..

[83] Breslin S., O’Driscoll L. (2016). The relevance of using 3D cell cultures, in addition to 2D monolayer cultures, when evaluating breast cancer drug sensitivity and resistance. Oncotarget.

[84] Lovitt C.J., Shelper T.B., Avery V.M. (2015). Evaluation of chemotherapeutics in a three-dimensional breast cancer model. J. Cancer Res. Clin. Oncol..

[85] Xu X., Farach-Carson M.C., Jia X. (2014). Three-dimensional in vitro tumor models for cancer research and drug evaluation. Biotechnol. Adv..

[86] Xu H., Liu W., Zhang X.Z., Hou L., Lu Y.J., Chen P.P., Zhang C., Feng D., Kong L., Wang X.L. (2016). Development of three-dimensional breast cancer cell culture drug resistance model. Sheng Li Xue Bao.

[87] Yildiz-Ozturk E., Gulce-Iz S., Anil M., Yesil-Celiktas O. (2017). Cytotoxic responses of carnosic acid and doxorubicin on breast cancer cells in butterfly-shaped microchips in comparison to 2D and 3D culture. Cytotechnology.

[88] Lovitt C.J., Shelper T.B., Avery V.M. (2018). Doxorubicin resistance in breast cancer cells is mediated by extracellular matrix proteins. BMC Cancer.

[89] Nunes A.S., Barros A.S., Costa E.C., Moreira A.F., Correia I.J. (2019). 3D tumor spheroids as in vitro models to mimic in vivo human solid tumors resistance to therapeutic drugs. Biotechnol. Bioeng..

[90] Wang R., Lv Q., Meng W., Tan Q., Zhang S., Mo X., Yang X. (2014). Comparison of mammosphere formation from breast cancer cell lines and primary breast tumors. J. Thorac. Dis..

[91] Yilmazer A. (2018). Evaluation of cancer stemness in breast cancer and glioblastoma spheroids in vitro. 3 Biotech.

[92] Chatterjee K., Zhang J., Honbo N., Karliner J.S. (2010). Doxorubicin cardiomyopathy. Cardiology.

[93] Koleini N., Kardami E. (2017). Autophagy and mitophagy in the context of doxorubicin-induced cardiotoxicity. Oncotarget.

[94] Li X., Lu Y., Liang K., Liu B., Fan Z. (2005). Differential responses to doxorubicin-induced phosphorylation and activation of Akt in human breast cancer cells. Breast Cancer Res..

[95] Smith L., Watson M.B., O’Kane S.L., Drew P.J., Lind M.J., Cawkwell L. (2006). The analysis of doxorubicin resistance in human breast cancer cells using antibody microarrays. Mol. Cancer Ther..

[96] Rivankar S. (2014). An overview of doxorubicin formulations in cancer therapy. J. Cancer Res. Ther..

[97] Vyas D., Laput G., Vyas A.K. (2014). Chemotherapy-enhanced inflammation may lead to the failure of therapy and metastasis. Onco Targets Ther..

[98] Zhong Z.F., Tan W., Tian K., Yu H., Qiang W.A., Wang Y.T. (2017). Combined effects of furanodiene and doxorubicin on the migration and invasion of MDA-MB-231 breast cancer cells in vitro. Oncol. Rep..

[99] Bandyopadhyay A., Wang L., Agyin J., Tang Y., Lin S., Yeh I.T., De K., Sun L.Z. (2010). Doxorubicin in combination with a small TGFbeta inhibitor: A potential novel therapy for metastatic breast cancer in mouse models. PLoS ONE.

[100] Liu C.L., Chen M.J., Lin J.C., Lin C.H., Huang W.C., Cheng S.P., Chen S.N., Chang Y.C. (2019). Doxorubicin Promotes Migration and Invasion of Breast Cancer Cells through the Upregulation of the RhoA/MLC Pathway. J. Breast Cancer.

[101] Li Q.Q., Xu J.D., Wang W.J., Cao X.X., Chen Q., Tang F., Chen Z.Q., Liu X.P., Xu Z.D. (2009). Twist1-mediated adriamycin-induced epithelial-mesenchymal transition relates to multidrug resistance and invasive potential in breast cancer cells. Clin. Cancer Res..

[102] Li Q., Ma Z., Liu Y., Kan X., Wang C., Su B., Li Y., Zhang Y., Wang P., Luo Y. (2016). Low doses of paclitaxel enhance liver metastasis of breast cancer cells in the mouse model. FEBS J..

