# Supplementary figures and images for: Spheroid Model of Mammary Tumor Cells: Epithelial–Mesenchymal Transition and Doxorubicin Response
Source: Biology (Basel). 2024 Jun 21;13(7):463. doi: 10.3390/biology13070463 (PMC11273983; doi:10.3390/biology13070463)

Figure S1: Figure shows western blotting in triplicate for E-cad (A) and VIM (B), related to Figure 8.

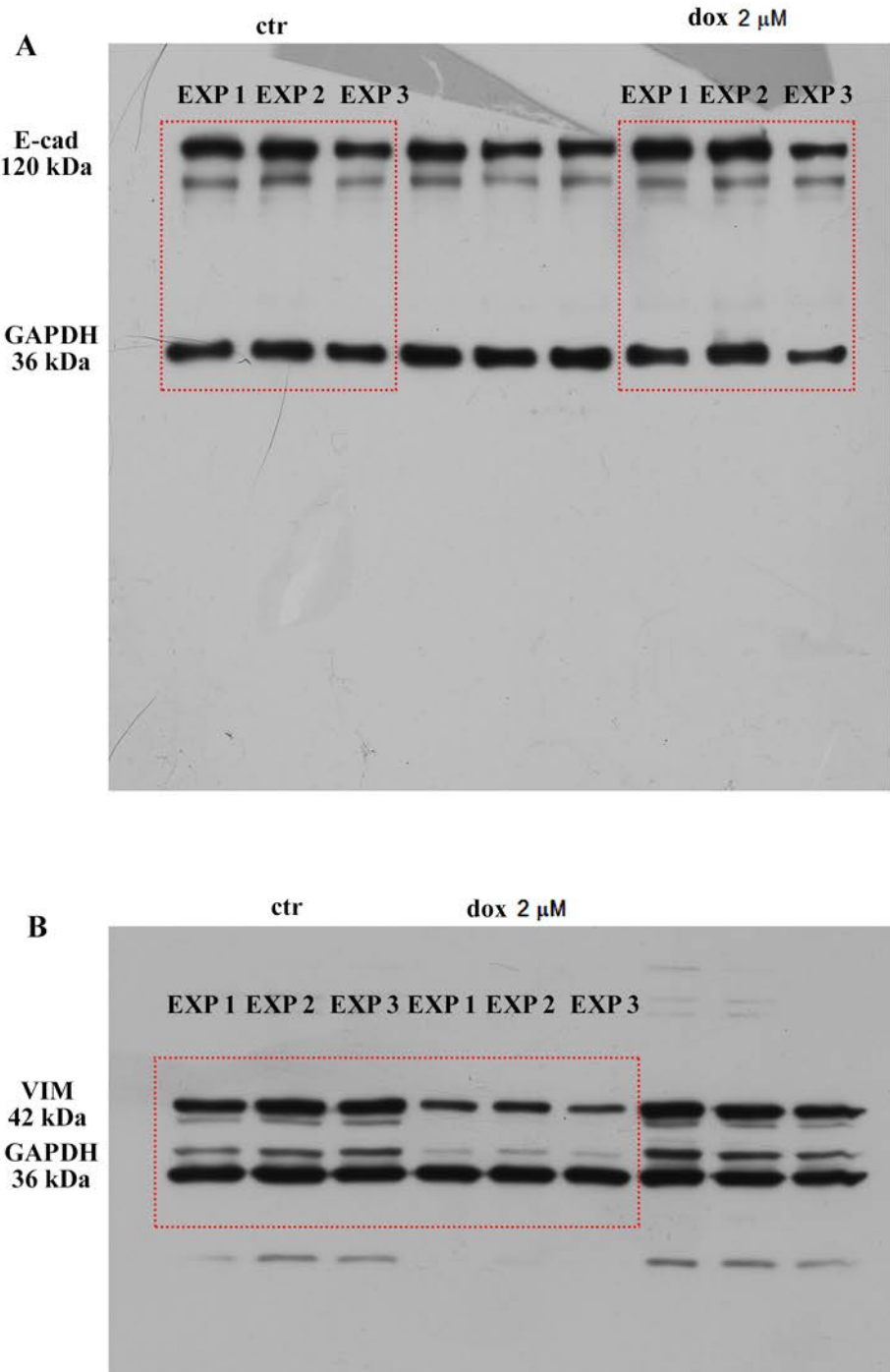

Supplement: Supplementary file 1 [file biology-13-00463-s001.zip › biology-2983348-supplementary.pdf]
